# Usefulness of right ventriculography compared with computed tomography for ruling out the possibility of lead perforation before lead extraction

**DOI:** 10.1371/journal.pone.0245502

**Published:** 2021-03-04

**Authors:** Saori Asada, Nobuhiro Nishii, Takayoshi Shinya, Akihito Miyoshi, Yoshimasa Morimoto, Masakazu Miyamoto, Koji Nakagawa, Kazufumi Nakamura, Hiroshi Morita, Hiroshi Ito

**Affiliations:** 1 Department of Cardiovascular Medicine, Okayama University Graduate School of Medicine, Dentistry and Pharmaceutical Sciences, Okayama city, Okayama, Japan; 2 Department of Cardiovascular Therapeutics, Okayama University Graduate School of Medicine, Dentistry and Pharmaceutical Sciences, Okayama city, Okayama, Japan; 3 Department of Pediatric Radiology, Okayama University Graduate School of Medicine, Dentistry and Pharmaceutical Sciences, Okayama city, Okayama, Japan; University Medical Center Groningen, University of Groningen, NETHERLANDS

## Abstract

**Purpose:**

High-risk patients can be identified by preprocedural computed tomography (CT) before lead extraction. However, CT evaluation may be difficult especially for lead tip identification due to artifacts in the leads. Selective right ventriculography (RVG) may enable preprocedural evaluation of lead perforation. We investigated the efficacy of RVG for identifying right ventricular (RV) lead perforation compared with CT in patients who underwent lead extraction.

**Methods:**

Ninety-five consecutive patients who were examined by thin-section non-ECG-gated multidetector CT and RVG before lead extraction were investigated retrospectively. Newly recognized pericardial effusion after lead extraction was used as a reference standard for lead perforation. We analyzed the prevalence of RV lead perforation diagnosed by each method. The difference in the detection rates of lead perforation by RVG and CT was evaluated.

**Results:**

Of the 115 RV leads in the 95 patients, lead perforation was diagnosed for 35 leads using CT, but the leads for 29 (83%) of those 35 leads diagnosed as lead perforation by CT were shown to be within the right ventricle by RVG. Three patients with 5 leads could not be evaluated by CT due to motion artifacts. The diagnostic accuracies of RVG and CT were significantly different (p < 0.001). There was no complication of pericardial effusion caused by RV lead extraction.

**Conclusion:**

RVG for identification of RV lead perforation leads to fewer false-positives compared to non-ECG-gated CT. However, even in cases in which lead perforation is diagnosed, most leads may be safely extracted by transvenous lead extraction.

## Introduction

In recent years, the number of cardiac implantable electronic device (CIED) implantations has increased, resulting in an increase in CIED complications such as infection, device malfunction, and lead breakage. Along with the increase in CIED implantations, cases of transvenous lead extraction are also increasing [1,2]. Computed tomography (CT) has been reported to be useful for identifying patients at risk of mechanical complications during lead extraction. It has been reported that perforation rates were 15% for atrial leads and 6% for ventricular leads in non-electrocardiogram (ECG)-gated CT [3]. Lewis et al. reported that ECG-gated multidetector CT (MDCT) is useful for excluding significant lead perforation and that MDCT suggestion of lead adhesion to central venous structures is associated with significantly longer laser times [4]. It is stated in guidelines that "ECG-gated cardiac CT is commonly used to identify ventricular lead perforation and appears more accurate [5]." However, especially for lead tip identification, CT evaluation is often difficult due to motion and severe streak artifacts of the leads. Reported event rates for lead perforation range from 0.1% to 0.8% for pacemaker leads and from 0.33% to 5.2% for implantable defibrillator leads [6,7]. Precise identification of the lead tip location is very important before lead extraction. If lead perforation is suspected, preparation for emergent surgery is necessary, or open-chest surgery may be employed to avoid cardiac tamponade in some cases. However, in many cases in which lead perforation was suspected by CT, open-chest surgery revealed the absence of perforation. Unnecessary invasive open-chest surgery should be avoided, especially in patients with infection. Right ventriculography (RVG) has been performed at the tip of the right ventricular (RV) lead in our hospital before lead extraction. RVG may be useful for evaluation of preprocedural lead perforation and ensure safe performance of the procedure. The aims of this study were to determine the accuracy of RVG for identifying RV lead perforation compared to the accuracy of thin-slice non-ECG-gated MDCT and determine the relationship between RV lead perforation diagnosed by each method and pericardial effusion during transvenous lead extraction.

## Materials and methods

### Subjects of the study

Among 145 patients who underwent lead extraction in Okayama University from July 2010 to March 2018, patients in whom lead extraction failed, patients who underwent only left ventricular (LV) lead or right atrial (RA) lead extraction, and patients who did not undergo preprocedural thin-slice MDCT or RVG were excluded. In the opinion of the surgeon and an experienced lead extraction expert, coronary angiography and RVG were necessary tests before lead extraction in our hospital except in cases of tricuspid valve or lead vegetation. We accessed the clinical data in May 2018. This study was a retrospective study on 115 RV leads in 95 patients that were successfully extracted using laser sheaths, mechanical sheaths, and snares. The primary reasons for performing lead extraction were lead infection (79 patients, including 99 leads), lead malfunction (10 patients, including 10 leads) and device upgrade (6 patients, including 6 leads). This study was approved by the Ethics Committee on Human Research and Epidemiology of Okayama University (project approval number: 1604–015) Informed consent of participants was not obtained since 1) this study was an observational study and 2) all of the data were analyzed anonymously.

### Pericardial effusion as a reference standard for lead perforation

Transesophageal echocardiography was used during the procedure to assist with lead localization and to provide clinically relevant information such as pericardial effusion. On the day of lead extraction, we followed up patients in the intensive care unit to make sure there was no hemodynamic disruption. The day after surgery, electrocardiography and transthoracic echocardiography were performed in all cases. Newly recognized pericardial effusion after lead extraction was used as a reference standard for lead perforation.

### CT imaging

CT was performed using a non-ECG-gated MDCT in all patients prior to lead extraction. There was variability in the CT protocol used by referring practitioners. Among 95 patients with 115 RV leads, contrast agents were not administered to 45 patients with 51 leads because of preexisting advanced renal disease or allergy to contrast agents. Slice thickness ranged from 0.5 to 3.0 mm, depending on the clinical indication. The average slice thickness of CT imaging was 1.30 ± 0.65 mm. To minimize streak artifacts of the leads, we used images of reconstructed orthogonal oblique multiplanar reformats (MPRs) from two directions to show the lead tip and those of a relatively wide window (~1400) with a center of approximately 300 housefield units (HU), equivalent to a modified bone setting [8]. In CT, lead perforation was defined as a situation in which the lead tip had passed through the epicardium.

### RVG imaging

**Fig 1:**
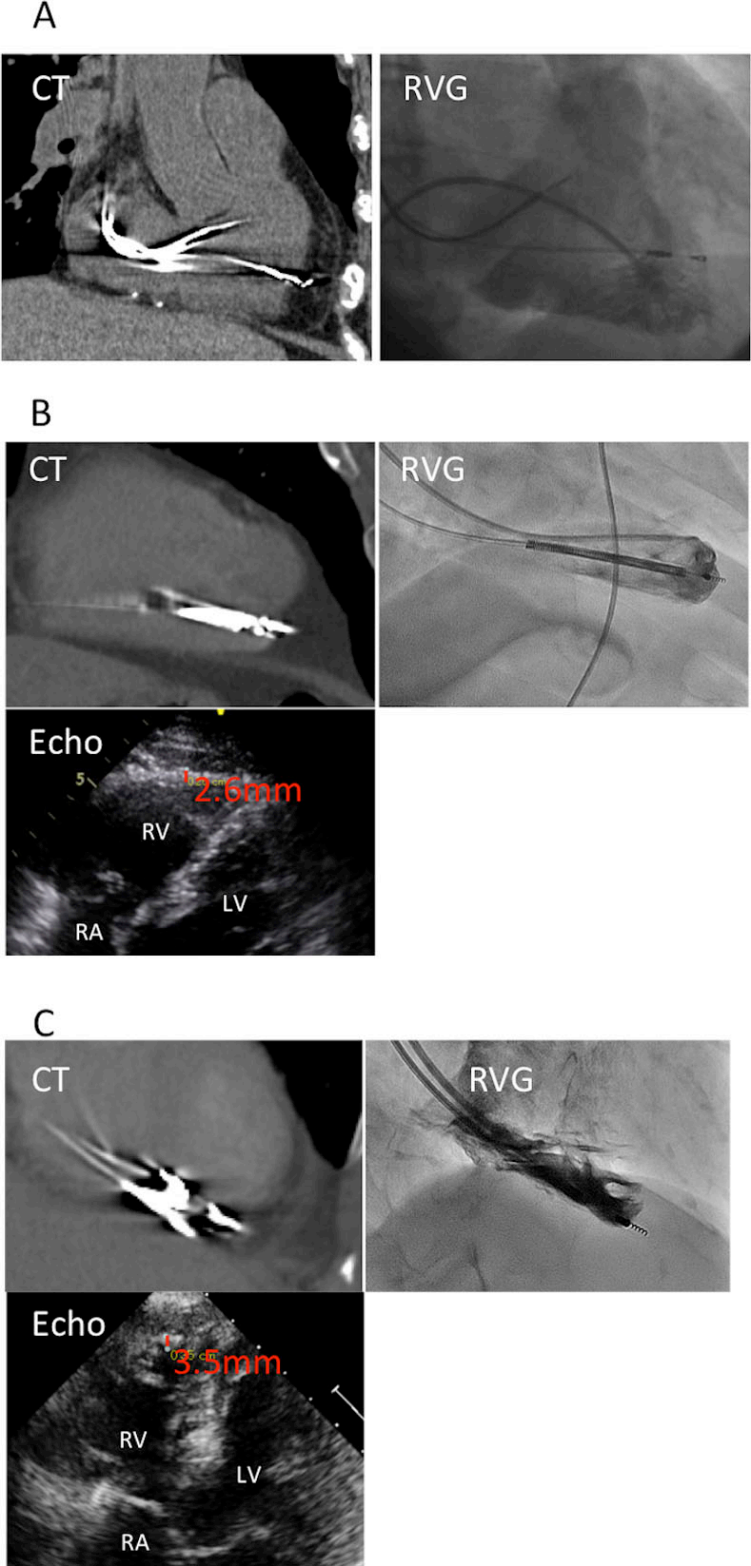
Panels A, B, and C show non-ECG-gated MDCT, RVG and echocardiography images, representatively, of a patient. (A) A diagnosis of lead perforation was made by MDCT but not by RVG. (B) Lead perforation was identified by MDCT; however, RVG showed that the lead tip was extruded 2.1 mm beyond the boundary of the cardiac chamber, and the right ventricle wall thickness obtained by echocardiography was 2.6 mm, indicating no lead perforation. (C) A diagnosis of lead perforation was made by RVG. The lead was difficult to evaluate due to motion artifacts in MDCT. MDCT, multidetector computed tomography; RVG, right ventriculography; echo, echocardiography; RV, right ventricle; RA, right atrium; LV, left ventricle.

RVG was performed in all patients prior to lead extraction. RVG was mainly selective RVG and sometimes full RVG with an injector and 40 mL of contrast medium (Fig 1). The cineradiography setting was selected for acquisition of 15 frames per second with a 10-inch full field of view. In selective RVG, approximately 10 mL of contrast medium was manually injected near the RV lead tip using a Berman angiographic catheter® (Teleflex Medical Japan, Ltd.). The RV lead tip was evaluated with a biplane 30-degree right anterior oblique view and a 50-degree left anterior oblique view. Lead perforation in RVG was evaluated using the two-step approach. In the first step, we determined whether the lead tip was in the cardiac chamber at the end of diastole. In the second step, if the lead tip had passed beyond the boundaries of the cardiac chambers, we measured the thickness of the RV free wall by echocardiography in an RV-focused view [9]. We defined lead perforation in RVG as perforation in which the lead tip had passed through the epicardium when the extrusion length of the lead tip beyond the cardiac chambers was longer than the thickness of the RV obtained by echocardiography.

### Image analysis

CT images were independently evaluated by one radiologist (T.S.) and one cardiologist (S.A.). RVG images were independently evaluated by two cardiologists (S.A. and N.N.). The three observers did not participate in the initial clinical interpretation of the images and did not know whether lead perforation was suspected. Consensus interpretations were performed in cases of disagreement. If the RV lead was diagnosed as lead perforation by each method, the length of extrusion beyond the epicardium was noted. In RVG, the extrusion length was the length of the lead tip beyond the endocardium minus the RV wall thickness obtained by echocardiography.

### Lead extraction procedure

The lead extraction procedure in all but 5 cases with 7 leads was performed under general anesthesia. Laser sheaths were used in all cases when the leads could not be explanted by traction alone. In brief, the lead was prepared by inserting a locking stylet into the inner coil lumen when possible. A suture was tied onto the insulation and locking stylet at two sites. Laser application was performed at the binding sites and advanced gradually from one binding site to another until the tip of the lead was reached. After abutting the myocardium, a combination of traction and counter-traction was performed, and the lead was freed. If laser sheaths could not be advanced, mechanical sheaths were used. A femoral or jugular approach was also attempted with snares [10].

### Statistical analysis

Continuous variables were summarized by using the means and standard deviation. Categorical variables were summarized as counts and proportions. In this study, pericardial effusion after right ventricular lead extraction was the reference standard for diagnosis of lead perforation, and we justified the accuracy of the results of CT and RVG. Differences in the detection of lead perforation between RVG and CT were analyzed using the McNemar test. A value of p < 0.05 was defined as statistically significant and all tests were 2-sided. All statistical analyses were performed using SPSS version 24.0 software (SPSS, Chicago, IL).

## Results

### Patient characteristics and extracted leads

Patient characteristics are shown in Table 1 and extracted lead characteristics are shown in Table 2. The mean age of the patients was 70.1 ± 14.8 years, and 70 patients (74%) were male. The mean body mass index of the patients was 22.4 ± 3.9 kg/m^2^ and the mean left ventricular ejection fraction was 57.9 ± 13.8%. Renal dysfunction (glomerular filtration rate < 60 mL/min/m^2^) was found in 46 patients (48%). Fourteen patients (15%) had prior sternotomy, 73 patients (77%) had pacemakers, 21 patients (22%) had implantable cardioverter defibrillators (ICD) and 8 patients (8%) had cardiac resynchronization devices (Table 1).

**Table 1:** Patient characteristics.

	N = 95
Age, years ± SD^a^	70.1 ± 14.8
Male, n (%)	70 (74%)
Body mass Index, kg/m^2^ ± SD	22.4 ± 3.9
NYHA^b^ class Ⅲ/Ⅳ, n (%)	11 (12%)
Diabetes mellitus, n (%)	25 (26%)
Estimated glomerular filtration rate < 60 mL/min/m^2^, n (%)	46 (48%)
Hypertension, n (%)	50 (53%)
Oral corticosteroid, n (%)	4 (4.2%)
Ejection fraction, % ± SD	57.9 ± 13.8
Implanted device, n (%)	
Pacemaker	73 (77%)
ICD^c^	14 (15%)
CRT-D^d^	7 (7%)
CRT-P^e^	1 (1%)
Prior sternotomy, n (%)	14 (15%)

^a^SD, standard deviation;

^b^NYHA, New York Heart Association;

^c^ICD, implantable cardiac-defibrillator;

^d^CRT-D, cardiac resynchronization therapy with a defibrillator;

^e^CRT-P, cardiac resynchronization therapy.

**Table 2:** Extracted lead characteristics.

	n = 115
Fixation type, n (%)	
Active	59 (51%)
Passive	56 (49%)
Right ventricle lead, n (%)	115 (100%)
Type of lead, n (%)	
PM-bipolar^a^	77 (67%)
PM-unipolar^b^	6 (5%)
PM-VDD^c^	5 (4%)
ICD-DC^d^	18 (16%)
ICD-SC^e^	9 (8%)
Dwelling time, years ± SD^f^	9.3 ± 7.2

^a^PM-bipolar, pacemaker bipolar lead;

^b^PM-unipolar, pacemaker unipolar lead;

^c^PM-VDD, pacemaker VDD lead;

^d^ICD-DC, dual-coil implantable cardiac-defibrillator lead;

^e^ICD-SC, single-coil implantable cardiac-defibrillator lead;

^f^SD, standard deviation.

Approximately half of the extracted leads were leads with active fixation (n = 59, 51%) and 56 (49%) were leads with passive fixation. Defibrillator leads accounted for 27 (24%) of the extracted leads and 18 (66%) of those leads were dual-coil leads. The average lead dwelling time was 9.3 ± 7.2 years (Table 2).

### Characteristics of cases diagnosed with lead perforation

Thirty-one patients with 37 leads were diagnosed as having lead perforation by CT or RVG. Ten (32%) of those patients were female, with a mean age of 70.3 years. Most of those leads (95%) were extracted due to indication of infection. Defibrillator leads accounted for 13 (35%) of those extracted leads, with 9 (69%) being dual-coil leads. Eighteen (48%) of the perforating leads were active fixation leads, and the remaining 19 (52%) were passive fixation leads. The average lead dwelling time was 10.1 years. Two leads (5%) showed abnormal pacing parameters, and 6 leads (16%) were abandoned leads (Table 3).

**Table 3:** Demographic features of individual cases diagnosed with perforation by CT or RVG.

Subject	Age	Sex	indication of lead extraction	perforating lead	fixation type	lead dwelling time	electrical parameters	lead perforation by CT^a^	lead perforation by RVG^b^
1	79	M^c^	infection	PM^d^-bipolar	passive	7.3	WNL^e^	+	-
2	60	F^f^	infection	PM-bipolar	passive	8.2	WNL	+	-
3	66	M	infection	PM-bipolar	passive	5.7	WNL	+	-
4	77	M	infection	PM-bipolar	passive	7.1	WNL	+	-
5	63	F	infection	PM-bipolar	passive	32.9	abandoned	+	-
			infection	PM-unipolar	passive	18.6	WNL	+	-
6	72	M	infection	PM-bipolar	active	4.1	WNL	+	-
7	66	M	infection	ICD-DC^g^	passive	8.1	WNL	+	-
8	69	M	infection	ICD-SC^h^	active	0.2	WNL	+	-
9	79	F	infection	PM-bipolar	active	14.2	WNL	high artifact (difficult to evaluate)	+
			infection	PM-bipolar	passive	22.0	abandoned	+	-
10	82	M	infection	PM-bipolar	active	1.8	WNL	+	-
11	76	F	infection	PM-bipolar	active	2.1	WNL	+	-
12	84	M	infection	PM-bipolar	active	12.5	WNL	+	-
13	80	M	infection	ICD-DC	passive	12.1	abandoned	+	-
			infection	ICD-SC	active	11.1	high impedance	+	+
14	84	M	infection	PM-bipolar	passive	4.6	WNL	+	-
15	42	M	infection	PM-bipolar	passive	26.9	WNL	+	-
			infection	PM-unipolar	passive	27.8	abandoned	+	-
16	48	M	infection	ICD-DC	active	11.5	WNL	+	+
17	74	M	infection	ICD-DC	active	6.5	WNL	+	-
18	72	F	infection	ICD-DC	active	5.0	WNL	+	-
19	84	F	infection	PM-bipolar	passive	11.4	WNL	+	-
20	80	F	infection	PM-bipolar	active	8.4	WNL	+	-
21	84	M	infection	PM-bipolar	passive	11.1	WNL	+	-
22	81	F	infection	PM-bipolar	passive	14.6	WNL	+	-
23	42	M	lead malfunction	ICD-DC	passive	6.6	low shock impedance	+	-
24	53	M	infection	ICD-DC	active	11.6	WNL	+	-
25	43	M	infection	ICD-DC	active	3.4	WNL	+	-
26	54	M	infection	ICD-SC	active	2.9	WNL	+	-
27	65	F	device upgrade	PM-bipolar	active	5.8	WNL	+	-
28	74	M	infection	ICD-SC	active	6.5	WNL	+	+
29	82	M	infection	ICD-DC	active	1.6	WNL	+	-
			infection	PM-bipolar	passive	14.9	abandoned	+	+
			infection	PM-VDD	passive	16.4	abandoned	+	-
30	85	M	infection	PM-bipolar	passive	0.7	WNL	high artifact (difficult to evaluate)	-
31	79	F	infection	PM-bipolar	active	6.6	WNL	+	+

^a^CT, computed tomography;

^b^RVG, right ventriculography;

^c^M, male;

^d^PM, pacemaker;

^e^WNL, within normal limits;

^f^F, female;

^g^ICD-DC, dual-coil implantable cardiac-defibrillator lead;

^h^ICD-SC, single-coil implantable cardiac-defibrillator lead.

### Image findings

Both RVG and CT were performed in all of the patients before lead extraction. No patients developed complications due to the RVG or CT. Of the 115 RV leads, lead perforation was observed for 35 leads by CT, but 25 of the 35 leads for which lead perforation was diagnosed by CT were shown by RVG to be within the RV chamber. Four leads were imbedded in the RV tissue according to the RV thickness obtained by echocardiography. Finally, for 29 (83%) of the 35 leads for which lead perforation was diagnosed by CT, RVG indicated that there was no perforation. Perforation was diagnosed by RVG for only 6 leads. All cases diagnosed as lead perforation by RVG were also diagnosed as lead perforation by CT, and among the patients who were not diagnosed as having lead perforation by RVG, none were diagnosed as lead perforation by CT. Three patients with 5 leads could not be evaluated by CT due to motion artifacts. There were no complications of pericardial effusion caused by RV lead extraction in any of the patients including patients who were suspected by CT or RVG of having lead perforation. The sensitivity of CT was incalculable and the specificity was 68.2% (Table 4) and the sensitivity of RVG was incalculable and the specificity was 94.8% when pericardial effusion after RV lead extraction was used as the reference standard (Table 5).

**Table 4:** Diagnosis of lead perforation by CT.

		Pericardial effusion after ^a^RV lead extraction	
		(+)	(-)
CT^b^	Lead perforation (+)	0	35
	Lead perforation (-)	0	75
		Sensitivity incalculable	Specificity: 68.2%

In CT, 3 patients with 5 leads were unable to be evaluated due to motion artifacts.

^a^RV lead, right ventricular lead;

^b^CT, computed tomography.

**Table 5:** Diagnosis of lead perforation by RVG.

		Pericardial effusion after RV lead^a^ extraction	
		(+)	(-)
RVG^b^	Lead perforation (+)	0	6
	Lead perforation (-)	0	109
		Sensitivity incalculable	Specificity: 94.8%

^a^RV lead, right ventricular lead;

^b^RVG, right ventriculography.

The difference between the rates of detection of lead perforation by RVG and CT was statistically significant (McNemar test, p < 0.001; Table 6).

**Table 6:** Diagnosis of lead perforation by RVG and CT.

		CT^a^	
		Lead perforation (-)	Lead perforation (+)
RVG^b^	Lead perforation (-)	75	29
	Lead perforation (+)	0	6

In CT, 3 patients with 5 leads were unable to be evaluated due to motion artifacts.

^a^CT, computed tomography;

^b^RVG, right ventriculography.

The difference was also statistically significant when we analyzed only cases of CT with contrast agents (McNemar test, p < 0.001; Table 7).

**Table 7:** Diagnosis of lead perforation by RVG and contrast-enhanced CT.

		CT^a^	
		Lead perforation (-)	Lead perforation (+)
RVG^b^	Lead perforation (-)	44	11
	Lead perforation (+)	0	5

^a^CT, computed tomography;

^b^RVG, right ventriculography.

Representative cases of both types of imaging are shown in Fig 1.

The maximum extrusion lengths of the lead tip were 5.4 mm in RVG (Fig 1, Panel C) and 9 mm in CT. The average RV free wall thickness in cases in which RVG showed that the lead tip had passed beyond the boundaries of the RV chambers was 3.2 ± 0.46 mm in echocardiography. The average extrusion length of the lead tip was significantly shorter in RVG than in CT (RVG vs. CT: 0.61 ± 0.72 vs. 3.1 ± 1.3 mm, p < 0.001). There was no significant interobserver difference in either CT or RVG in the diagnosis of perforation vs non-perforation or in identifying the average extrusion length of the perforating lead.

### Procedural outcomes

Of the 115 RV leads, 9 leads were extracted by simple traction, 48 were extracted by using only a laser sheath, and 37 were extracted by using both a laser sheath and mechanical sheath. A femoral approach was tried in 21 RV leads. Lead extraction was achieved in all cases. In all cases in this study, there was no major complication related to RV lead extraction, no hospital death, and no death within 30 days.

## Discussion

### New observations

Based on the results of the current study, there are two important clinical implications. First, RVG was more useful than non-ECG-gated MDCT for ruling out the possibility of lead perforation, and RVG may therefore reduce the rate of unnecessary open-chest surgery. Second, even in cases in which lead perforation up to 5.4 mm was diagnosed by RVG, transvenous lead extraction may be safely performed.

### Evaluation of the roles of RVG and CT before lead extraction

Precise identification of the lead tip prior to lead extraction is very important. If lead perforation is suspected, especially in cases in which the perforating lead is a tined lead or ICD lead or in cases in which the duration of perforation has been long, preparation for emergent open-chest surgery may be needed to avoid cardiac tamponade in some cases. However, even when lead perforation is suspected following CT assessment, open-chest surgery often reveals that there is no perforation. Unnecessary invasive open-chest surgery should be avoided to reduce the risk of additional infection of the mediastinum, pericardium and intrathoracic space.

A definitive diagnosis of lead perforation can be established by using thoracoscopy or sternotomy [11]. However, these procedures are invasive and thus difficult to perform in all cases, leaving imaging evaluation by radiography, echocardiography, and CT as the common modalities of assessment [12]. There have been several studies in which chest radiographs, echocardiography and CT were compared. Of these three imaging modalities, CT has been the gold standard for the diagnosis of lead perforations [5,8,12–14]. CT is an excellent tool for evaluating venous stenosis or occlusion, abnormal anatomical findings, and vascular adhesion prior to lead extraction [4]. On the other hand, CT may be more prone to false positives as a diagnostic method, especially for identifying RV lead perforation. For example, Hirschl et al. reported the prevalence of asymptomatic pacemaker and ICD leads on CT, in which 15% (15 of 100) of the patients had a lead perforation; however, none of the leads except for one ventricular lead showed abnormal pacing parameters [3]. Although the CT was not ECG-gated in their study, this finding suggests a risk of over-diagnosing perforation using CT scans, because most of the studies on perforation cases showed abnormal pacing parameters [11,13]. Recently, ECG-gated MDCT has been recommended as it has the potential for improved accuracy in diagnosing lead perforation compared to non-ECG-gated MDCT. However, lead artifacts still remain an impediment to the accuracy of determination of intravascular lead positioning [12,14]. This study showed that 83% of the lead perforations diagnosed by non-ECG-gated MDCT were excluded by RVG and that there were no leads that could not be evaluated due to motion artifacts. RVG was performed safely in all patients, and there were no complications associated with this procedure. These findings suggest that RVG may be more accurate than CT for ruling out the possibility of RV lead perforation. One of the drawbacks of RVG is that the RV wall thickness cannot be measured. Instead, we measured RV wall thickness by using echocardiography. The right ventricle has a pitted uneven surface. Although the wall thickness evaluated by echocardiography does not always match the wall thickness at the lead tip, it is useful as a guide for wall thickness. This technique may not be practical in real time, but it is useful in preoperative evaluations. Therefore, we propose that RVG is an appropriate method for RV lead tip evaluation.

### Lead extraction

In the present study, there were no complications of pericardial effusion caused by transvenous RV lead extraction in cases in which lead perforation was suspected. There are two possible reasons for this: false-positive imaging or safety of lead extraction even if lead perforation has occurred. In the Real-World Experience With National Cardiovascular Data Registry, the incidence of cardiac perforation was reported to be 0.35%; however, cardiac perforation was not associated with an increased risk of complications in multivariable logistic regression analysis [2]. In addition, many previous studies have suggested that lead extraction can be performed percutaneously even in cases in which lead perforation has been diagnosed [13,15,16]. Some previous studies have suggested that there are “self-sealing” properties by which the myocardium constricts and covers the perforated site just after lead traction, thus preventing pericardial effusion [17,18]. In addition, the most chronically perforated leads might migrate through the myocardium and invoke sufficient adhesion to seal the lead from the rest of the pericardium. On the other hand, cardiac tamponade following lead extraction of a perforating lead has been reported [4,18]. Although cases in which cardiac tamponade occurs are relatively rare, especially when perforation is suspected, it is necessary to prepare for emergent thoracotomy and perform percutaneous lead extraction. More accurate prediction at the stage of preoperative image evaluation is also important. Only the RV lead tip was evaluated in this study; however, myocardial or venous intrapericardial injury can occur anywhere along the lead body. Therefore, when extracting the lead, it is necessary to pay attention not only to evaluation of the lead tip but also to evaluation of lead adhesion or venous occlusion in clinical practice. It was difficult to determine whether newly recognized pericardial effusion was due to extraction of the perforated lead or due to the extraction procedure, but in this study, there was fortunately no pericardial effusion associated with lead extraction. We could therefore use pericardial effusion as a reference standard.

### Limitations

This study has some limitations. First, our results and conclusions are based on a limited number of cases in a single-center analysis. A larger multicenter prospective study is required to confirm the usefulness of RVG for diagnosis of lead perforation. Second, we retrospectively analyzed only the standard comprehensive thin-slice non-ECG-gated MDCT for evaluating cardiac perforation because of its clinical accessibility, and there was heterogeneity in the protocols used. This may explain why the rate of diagnosis of perforations in this study was higher than that in previous studies. A future study in which CT is prospectively performed with ECG gating or a program with a few lead artifacts may provide more reliable results. Third, regarding the evaluation by RVG, it is desirable to evaluate RVG from more directions for accurate diagnosis, but it is clinically difficult due to the large amount of contrast medium used, so we evaluated only in two directions. In addition, there is a possibility of creating a bias to a more specific result by using echocardiography, but we used this technique as it is difficult to measure the RV wall thickness using RVG alone. Fourth, a definitive diagnosis of lead perforation requires thoracoscopy or sternotomy, but it is invasive and difficult to perform in all cases. Therefore, pericardial effusion after RV lead extraction was used as the reference standard for diagnosis of lead perforation in this study. However, there are cases in which the lead can be extracted percutaneously without the occurrence of pericardial effusion even in cases in which lead perforation has been diagnosed.

## Conclusion

The use of RVG for identification of lead perforation is a diagnostic method that yields less false-positive results than those yielded by thin-slice non-ECG-gated MDCT. We propose that RVG is more useful than non-ECG-gated MDCT for ruling out the possibility of RV lead perforation. Special caution is needed in cases in which lead perforation has been diagnosed, but most leads may be extracted safely by transvenous lead extraction.
